# The Bamberg Trucking Game: A Paradigm for Assessing the Detection of Win–Win Solutions in a Potential Conflict Scenario

**DOI:** 10.3389/fpsyg.2018.00138

**Published:** 2018-02-13

**Authors:** Dario Nalis, Astrid Schütz, Alexander Pastukhov

**Affiliations:** Department of Psychology, University of Bamberg, Bamberg, Germany

**Keywords:** assessment tool, paradigm for cooperation, method development, win–win solutions, actor and partner effects, negotiation, fixed-pie bias

## Abstract

In win–win solutions, all parties benefit more from the solution than they would if they each pursued their own individual goals. Such solutions are beneficial at individual and collective levels and thus represent optimal solutions. Win–win solutions are desirable but often difficult to find. To allow the study of individual differences and situational factors that help or hinder the detection of win–win solutions, we created a paradigm that fills a gap in the repertoire of psychological instruments used to assess collaboration, cooperation, negotiation, and prosocial behavior. The new paradigm differs from previous ones in two aspects: (a) In existing paradigms that focus on social motivation, possible strategies are evident, whereas we focused here on the question of whether people can detect the solution and thus disentangle ability from motivation, (b) Paradigms that focus on cooperation typically entail a risk associated with the partner’s defection, whereas cooperation in our paradigm is not associated with risk. We adjusted the Trucking Game—a method for assessing bargaining—to include a situation in which two parties can help each other achieve their respective goals and thus benefit over and above the pursuit of individual goals or compromising. We tested scenario-based and interaction-based versions with samples of 154 and 112 participants, respectively. Almost one third of the participants or dyads found the win–win solution. General mental abilities were not related to detecting the win–win solution in either version. The paradigm provides a way to extend research on cooperation and conflict and can thus be useful for research and training.

## Introduction

The success of human civilizations has always largely depended on the cooperation of its citizens. Cooperative endeavors entail the potential to achieve more than individual efforts can alone. When the outcome of such cooperation exceeds the outcomes of individual efforts for each participating party, it is called a win–win solution. Win–win solutions, as they are beneficial for everyone participating, are thus extremely valuable achievements. Still, the possibility of achieving such a solution is not always obvious. In many cases, as exemplified in mediation and conflict resolution trainings (e.g., Kestner and Ray, 2002), people assume that they have to compete with the other party to achieve their own goals. In other words, they regard an open or ambiguous situation as a zero-sum situation (Rozycka-Tran et al., 2015) or conflict scenario. As mediation practice shows, however, in many cases, it is possible to reframe the situation and see the potential to collaborate and thus increase one’s own benefit as well as the other parties benefit. In other words, a situation that may be regarded as a zero-sum game often can be reframed to be understood as a win–win situation. In the present study, we created a paradigm that can be used to assess whether people are able to detect win–win solutions. The tool can be used for research that is interested in finding out which situational factors support or interfere with the finding of such solutions and which personality traits are linked to the probability of finding such solutions. We present a scenario-based version and an interaction-based computer simulation of this paradigm and show that the tool is not simply a mind puzzle as solutions are unrelated to general mental abilities.

### Conflict and Win–Win Solutions

Human interactions are often conceptualized as consisting of individual actors who have individual goals or interests. When two or more individuals act together with the aim of achieving a shared goal, this behavior is termed cooperation (Argyle, 1991). Sometimes, however, individual goals appear to be incompatible, and actors have the impression that someone else’s gains inevitably mean their own losses. In other words, situations are perceived as *zero-sum* scenarios (Von Neumann and Morgenstern, 1945; Rozycka-Tran et al., 2015). In research on negation, this belief in a zero-sum world has been called a *fixed-pie bias*, that is, negotiators believe that the goal is to distribute a fixed amount of goods and that every gain for one party simultaneously means a loss for the other party. Negotiators with a fixed-pie bias systematically fail to achieve optimal distributions because they do not even look for win–win solutions but are satisfied with mere compromise (Bazerman, 1983; De Dreu et al., 2000; Harinck et al., 2000). Such perceptions can be erroneous, meaning there is a possible solution but people just do not see it. Thus it is important to find out which traits and which situational factors facilitate the finding of such solutions.

Situations perceived as zero-sum situations establish the basis for conflict because participants are under the impression that they have to compete with the other person in order to achieve their own goals. As Wallensteen (2015) elaborated, a conflict is a situation in which “(…) a minimum of two actors (parties) strive to acquire at the same moment in time an available set of scarce resources” (p. 17f). Thus, detecting win–win solutions also means detecting solutions to potential or actual conflict. In fact, win–win solutions make conflict obsolete as they dissolve its basis. If actors can achieve more together than they could by pursuing their own individual goals, it no longer makes sense to compete as resources are no longer considered scarce. Thus, in interpersonal conflict and negotiation, discovering and implementing win–win solutions can be considered the optimal way to transcend conflict (Ury et al., 1988; Galtung, 2004).

We define win–win solutions *as outcomes of interpersonal behavior that exceed the outcomes that each participant could achieve alone.* Thus, win–win situations are valuable both individually and collectively. It is important to differentiate between a win–win solution and a compromise. Even though a compromise may be viewed as an acceptable solution to a conflict, parties that compromise achieve an outcome that is below their individual goals (Pruitt and Carnevale, 1993).

### Previous Paradigms to Study Cooperation with a Focus on Motivation

Studies on cooperation often aim to analyze prosocial behavior (Argyle, 1991; Van Lange et al., 2013). The paradigms that are used often include dilemmas in which one party’s gains are associated with another party’s losses (zero-sum games) to study prosocial attitudes. The extent to which actors are willing to give up their own benefit in favor of another actor’s benefit is often used as a measure of prosociality or altruism. In such paradigms prosocial behavior means the sacrifice of self-interest, e.g., the dictator game (Kahneman et al., 1986; Engel, 2011; Leder and Schütz, 2018) and the slider measure of social value orientation (Murphy et al., 2011). In both of these paradigms, participants are asked to distribute goods between themselves and another party. Prosociality is thus operationalized as the amount of money a person gives to the partner instead of keeping it for himself or herself. One variation of the dictator game is the ultimatum game (Güth et al., 1982; van’t Wout and Leder, 2018), in which the receiver can decide whether he accepts the offer or not. If he rejects, neither of the parties receives anything. Here the reason for giving resources to the other player is not only prosocial but also serves the self-interest of decreasing the probability of leaving empty handed. However, in both variations the maximal payoff cannot be increased by cooperation because there is no win–win solution to the game. In contrast, we aimed at designing a paradigm in which both individual and overall payoffs can be maximized.

Similarly, in public goods games (Hardin, 1968; Keil et al., 2016), the measure of prosocial behavior is the amount of resources a participant donates for the public good. Here multiple players decide on how much resources they want to donate to a shared pool. The pool then gets multiplied and the resources are evenly distributed among all players. The games are designed in such a way that it is profitable for individuals to free-ride as long as others behave in a prosocial manner, which means that defecting provides optimal solutions when the partners cooperate. In the prisoner’s dilemma (Rapoport and Chammah, 1965) two players must decide whether they confess a crime or stay mum. If both players stay mum, they both get a small punishment, if both confess, they both get a medium sized punishment, however, if one players stays mum and the other confesses, the first players receives a large punishment, while the second player receives no punishment at all. Staying mum is sometimes considered the same as being cooperative. Still, betraying a cooperative interaction partner again involves advantages to the individual. In other words, these paradigms intertwine prosocial behavior with the risk of the partner defecting and lack of prosocial behavior with personal benefit.

All of these paradigms are about motivation, i.e., the prosocial option is evident and the question is whether participants choose it. In contrast, our paradigm is not about motivation but about ability: it provides an ambiguous situation and tests whether a non-obvious win–win solution can be detected. Thus, there is not a dichotomy of self-interest versus other-interest, which means the paradigm disentangles the ability to find win–win solutions from motivational factors: win–win solutions are to be preferred by both selfish and altruistic actors because such solutions maximize each person’s benefit as well as the overall benefit, thus disentangling prosociality and self-sacrifice.

In the stag hunt game (Skyrms, 2001), the cooperative solution to the game is actually a win–win solution because both the collective and the individual payoffs are maximized. Two players decide whether they want to hunt a rabbit individually or cooperate with each other to hunt a stag. If they chose to hunt the rabbit, they are certain to slay the rabbit, which is a small gain. If they chose to hunt the stag, they can only slay it if both players chose to hunt the stag. In this paradigm, the win–win solution comes with a risk—if the interaction partner does not comply with the cooperative strategy (hunting the stag), the cooperative person is left empty handed. Therefore, whereas in the dictator and public goods games, prosocial behavior is confounded with a readiness to sacrifice individual goals, in the stag hunt game, the win–win solution is confounded with a willingness to take risks, also a motivational aspect. In the paradigm we present here, the win–win solution does not entail any risk. Finally, what all these games have in common is that the participants know the payoffs in advance. In real-world conflicts, payoffs as well as possible solutions are often unknown, however. In the present paradigm likewise possible pay-offs are unknown in an attempt to increase its external validity.

Studies on negotiations often use tasks in which the items have different values that depend on the role (e.g., buyer or seller) assigned to the participant (e.g., Pruitt and Lewis, 1975). The different values allow for an optimal distribution of items in order to maximize the joint utility for the negotiating parties. Trötschel et al. (2011) used a negotiation task in which the participants assumed the roles of spouses in a divorce. They negotiated with respect to nine items that each had different values for each partner. There was an optimal item distribution that depended on the value each spouse personally assigned to each item. Whereas these tasks aim at finding solutions that are as good as possible, they do not model win–win solutions because individual parties would still benefit from keeping all the items for themselves, even though it would not be realistic to expect such an outcome. In these negotiations, the aim is to achieve a pareto-optimal distribution of items, meaning that the utility for one actor is maximized, provided that doing so does not reduce the other actor’s utility. Also, the tasks arguably require a high capacity for reasoning and problem solving, and participants often have difficulty understanding the situation. Thus, our aim was to design a paradigm that would be easy to understand and would entail a true win–win solution. A typical win–win situation in everyday life can be considered the division of labor. Because everyone does only the job they specialize in, everyone involved produces more than he or she would have produced alone.

### A New Paradigm for Assessing the Detection of Win–Win Solutions

Because the detection of win–win solutions is very valuable in terms of optimal collaboration, it is arguably a worthwhile endeavor for psychologists to study conditions and traits that facilitate or hinder the discovery of win–win solutions. The scientific study of this issue requires research methods that accurately model social situations in which win–win solutions can be found, irrespective of prosocial motivation or willingness to accept risks. A method that could be applied to assess whether people are *able* to find win–win solutions, even when such solutions exist but are not obvious, had yet to be designed. We aimed to design a paradigm for assessing the detection of win–win solutions unconfounded with other variables (e.g., sacrifice of self-interest, willingness to trust others, or risk-aversion). The paradigm would need to be aimed at assessing the ability to see win–win solutions in situations that could also be perceived as potential conflict, i.e., involving conflicts of interest. The method would need to fulfill the following criteria:

(1) Individual gains for both parties applying the win–win solution exceed all possible individual gains from applying any other strategies.
(2) The individual gain for each party using the win–win solution exceeds the individual gain derived from a scenario in which the other party was non-existent.
(3) Non-compliance of Party A in the win–win solution is not associated with costs to Party B if Party B goes through with the win–win strategy as compared with any different strategy.
(4) The win–win solution is not associated with risks for the individual parties.
(5) One party does not benefit from non-compliance when the other party pursues the win–win solution. This means that there is no option to derive benefits from free-riding or defecting.
(6) The win–win solution is not obvious. The discovery of the win–win solution is an accomplishment. Operationally, this means that only some participants will detect the win–win solution.

## Development of the Bamberg Trucking Game

### The Original Trucking Game

We used the Trucking Game by Deutsch and Krauss (1962) as a blueprint and adapted it to meet the requirements above. The original Trucking Game served as a method for assessing bargaining behavior in social conflict. Participants were told to imagine they were operating a trucking company and needed to transport merchandise from one spot to another as efficiently as possible. Fictitious money was spent in relation to distance driven, which meant that the aim was to deliver goods via the shortest possible route. The situation included an aspect that provoked conflict: Another trucking company had similar goals, but their starting point was close to the other player’s end point, and their end point was close to the first player’s starting point. There was a short single-lane road that connected the starting and end points. It was impossible for the trucks to pass each other on this road, which created an incompatibility in goals and thus conflict. The short road was the scarce resource the two players competed for. There were also two longer private roads that the trucks could use, but these avenues were costly due to their longer distances. A typical compromise would be to find an agreement about when each company would use the shorter road.

Still, such a compromise is not a win–win solution as defined above: Criteria 2, 3, 4, and 5 are violated. Criterion 2 is the most important because it is associated with the design of the game itself. The scenario described above is designed in such a way that win–win solutions are not possible; there is no option that leads to a superior outcome for both players as compared with a scenario in which the other player was non-existent. If there was only one player, this player would be able to consistently use the short road: The existence of the other player is the very reason that each player cannot use the individually optimal option. Thus, again, the scenario is designed to involve conflict. Criterion 3 is violated because one player could allow the other player to use the short road every second turn, but if the other player does not allow the first player to use it in return, the first player would be better off trying to compete for the short road in every turn. This is related to the violation of Criterion 4. It is risky to pursue the compromise because one must depend on the other player complying with that strategy: Trust can be betrayed. Criterion 5 is violated because one player can benefit from defecting if the other player does not retaliate. If one player tries to use the short road even if it is not his or her turn, and the other player does not retaliate by doing the same thing, the defecting party benefits from having a go when it is not his or her turn. As elaborated above, the classic Trucking Game involves conflict, and the aim is thus to bargain to find a compromise. In order to satisfy our goal to design a method for assessing the detection of win–win solutions, the original trucking game had to be modified to fit the six criteria listed above.

### Modification of the Trucking Game to Assess the Detection of Win–Win Solutions

The original trucking game by Deutsch and Krauss (1962) did not include the possibility of a win–win solution but allowed only for a compromise. We thus included the possibility that each trucker would help each other out, that is, they would help each other to achieve better outcomes together than would be possible individually. For both players, it was possible to pick up the other player’s load and deliver it to that person’s destination. Of course, this information was not provided explicitly because our goal was to find out whether the players would detect this win–win solution.

**FIGURE 1:**
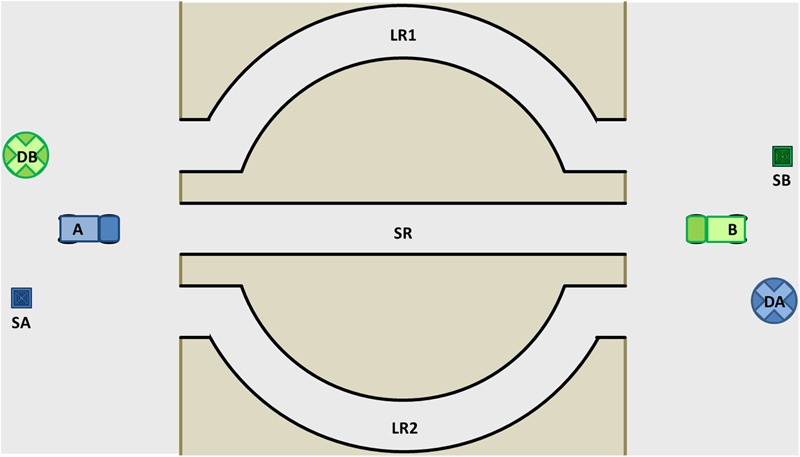
Constellation of the Bamberg Trucking Game. A, Truck Player A; B, Truck Player B; SA, Start Player A; SB, Start Player B; DA, Delivery point Player A; DB, Delivery point Player B; SR, Short road; LR1 and LR2, Long roads.

**Figure 1** shows the modifications to the original trucking game. There is still a short single-lane road (SR) and two longer roads (∼50% longer), LR1 and LR2, connecting the starting (SA and SB) and delivery (DA and DB) points of the players. In our modification, however, Player A can pick up Player B’s boxes (SB), and Player B can pick up Player A’s boxes (SA). Thus, both players can, without any further effort and hardly any loss of time, after delivering their own boxes at their own delivery points (DA and DB), pick up the other player’s box, and on their way back to their own starting points (SA and SB), drop them at the other player’s delivery point. If both players help each other to accomplish their respective tasks, the sum of delivered boxes will more or less double what would be the result of the classical compromise in the original trucking game and thus constitute a win–win situation. Furthermore, this option is also far better than what one player can achieve when playing alone. The purpose of our adaptation of the Trucking Game is exactly that: to assess whether participants discover this opportunity to engage in a mutually beneficial interaction.

To maximize the number of delivered loadings, the drivers need to agree to collaborate: truck A uses SR on the first round while truck B uses LR1 or LR2. When truck A has delivered the first round of goods at DA, truck B will have passed two thirds of the distance of LR. Truck A picks B’s loadings and uses SR again. By the time truck B has delivered his first round of goods and picked up A’s loading, truck A will have crossed half the distance of SR. Truck B uses LR once more on its second round and delivers A’s loadings. Both trucks will be on the same side after Truck A has delivered the third load and truck B has delivered the second load. For the rest of the time both trucks use only SR driving behind each other and delivering both their own goods and the other player’s goods.

We could have designed the scenario to be conflict free by replacing the single-lane short road (SR) with a two-lane short road, and we could still have assessed whether the participants would discover the win–win solution. However, we decided to keep the potential conflict issue from the original Trucking Game. This served a twofold purpose: (a) diverting participants’ attention from the win–win solution and (b) making the solution not only a way of cooperating for mutual benefit but also a means for transcending the would-be conflict (Galtung, 2004). In other words, at first sight, the scenario appears to look like a conflict in which SR is a scarce resource, but once the win–win solution is discovered, the conflict disappears because the players are no longer concerned about which player drives on the SR as both players transport each other’s goods. This situation seems typical of many conflicts in everyday life, in both the workplace and private life: A situation appears to be a zero-sum game but can ideally be transformed into a win–win solution. In fact, solutions in conflict resolution or mediation typically aim at just that: finding a win–win solution in a situation that at first sight looks like a conflict that offers a compromise at best.

## The Present Studies

The Bamberg Trucking Game is a tool for testing whether people are able to find win–win solutions. We created two versions: One is scenario-based, in which individual participants are presented with the description of the scenario and are asked to describe the strategy they would use to maximally benefit their company. The second is an interaction-based computer application in which two participants interact with each other, with each of them “driving” their own truck. We conducted two studies in which we tested the properties of each version. Our primary aim was to test the difficulty of the Bamberg Trucking Game and its practicability in each version. A secondary aim was to test discriminative validity with general mental ability.

## Study 1

The main aim of our first study was to investigate how difficult it would be for participants to discover the win–win solution in a scenario-based version of the Bamberg Trucking Game. The optimum level of difficulty for a single dichotomous outcome is 0.5 so that an equal number of participants find the solution and fail to find the solution. Apparently, the difficulty of 0.5 is the best choice when the ultimate goal is to investigate how traits and situational variables affect detection of the solution by means of logistic regression as the variability of the outcome variable is maximized at this level.

A second aim was to determine the percentage of participants who think about the win–win solution but do not suggest it because they consider it incompatible with the rules of the game. In early tests of the paradigm, we noted that some participants believed that the designers of the study did not intend the win–win solution, and thus, these participants regarded this cooperative strategy as a way of cheating in the game. We tested this possibility by asking the participants afterward about this possibility.

We also tested whether detecting the win–win solution was simply a matter of mental ability and related the outcome to a measure of intelligence. One way of looking at the scenario-based version of the Bamberg Trucking Game is to regard it as a cognitive puzzle akin to items on an intelligence test. The participant is confronted with a problem and is asked to provide the best possible solution. Mental abilities could thus help to solve the problem. For the tool to have divergent validity, the relationship between the two variables should be only small.

### Method

#### Sample

A total of 158 participants with an age range from 17 to 56 years (*M* = 25.7 years, *SD* = 6.11; 69.1% female) were recruited online via social and private networks to participate in our study; 39.9% were undergraduate students of which 35.2% were psychology majors. The study was advertised as a study on social attitudes and problem solving. We offered participants feedback on their performance on a cognitive task as incentive.

#### Instructions and Setup

The study was implemented online via the software package EFS Survey (Questback GmbH, 2016). Participants were told that the study would last approximately 25 min and that, at the end, they could receive feedback on their performance in a cognitive task.

Participants completed a measure of general mental ability, the Bamberg Trucking Game (scenario-based; see **Appendix A** for the English translation of the instructions), and additional questions. In the Bamberg Trucking Game, participants were asked to note the strategy they would use in a text box, phrased as instructions to their truck driver^1^. On the next page, participants were asked to write down ideas they thought about initially but considered not permissible. **Appendix B** shows the English translation of these instructions.

As a measure of mental ability, participants completed the Mini-Q (Baudson and Preckel, 2015), a 3-min intelligence screening test based on a verbal reasoning test developed by Baddeley (1968). On this test, participants are asked to decide as quickly as possible if a statement correctly describes a constellation of three (geometrical) figures by clicking a right or a wrong button next to the statement. The test contained four practice trials and 64 test trials. Intelligence scores were calculated for each participant by calculating the number of correct decisions. Internal consistency was high (α = 0.97).

Age and gender were collected as demographic information, and as a control question, participants were asked if they had already heard about the Trucking Game, and if so, what they knew about it. Finally, participants were asked whether they wanted feedback on the test of general mental ability.

### Results

**FIGURE 2:**
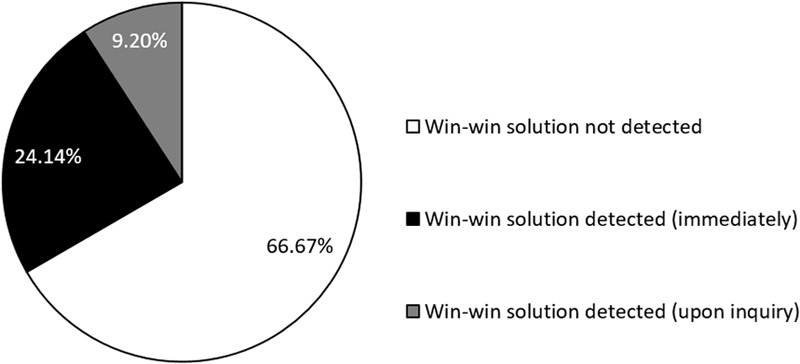
Distribution of categories of solutions in the Bamberg Trucking Game.

We analyzed the text written down by participants and coded *win–win solution detected* if a participant suggested that the truck drivers help each other by transporting each other’s goods on their way back. If the win–win solution was not mentioned on the first page, we further coded whether participants had mentioned helping each other on the next page, where they had been asked to state strategies that they had not yet stated on the prior page. A total of 66.7% of all participants did not mention the win–win solution on either of the pages, 24.1% provided the win–win solution on the first page, and 9.2% mentioned it upon further inquiry on the next page (see **Figure 2**).

We computed multiple logistic regression analyses to investigate how the demographic variables and intelligence predicted the detection of the win–win solution. To create one dichotomous dependent variable, the categories *immediate detection* and *detection upon inquiry* were collapsed into one category. Another reason for collapsing the two categories was that our aim was to measure the detection of win–win solutions. Participants who reported the solution upon inquiry did in fact see the solution, and there was no methodological reason to treat them differently than those who stated it right away. We entered age, gender, and the Mini-Q score (*M* = 37.24, *SD* = 11.59) as predictors into a multiple logistic regression analysis. The omnibus test of model coefficients was not significant (*χ*^2^ = 2.50, *p* = 0.47). As **Table 1** shows, none of the predictors significantly predicted the detection of the win–win solution.

**Table 1:** Logistic regression of the detection of the win–win solution on age, gender, and intelligence.

Predictor	*B*	*SE*	*Wald*	*p*
Age	0.001	0.02	1.71	0.19
Male	0.33	0.38	0.74	0.39
Mini-Q score	0.02	0.03	0.002	0.96

### Discussion

Detection of the win–win solution in the scenario-based version of the Bamberg Trucking Game proved to be rather difficult. Only one quarter of all participants found the solution when presented with the scenario. This number increased to one third if we included participants who mentioned the win–win solution after being asked to state strategies they thought were not permissible. A solution rate of one third seems satisfactory for justifying the use of the Bamberg Trucking Game results as the dependent variable in the logistic regression analyses.

The Mini-Q test of GMA (Baudson and Preckel, 2015) did not predict whether participants found the win–win solution, suggesting that the task is not simply about cognitive abilities. Instead, detecting win–win solutions may require a certain mind-set and not general mental abilities (e.g., creative problem solving). To be sure, the Mini-Q is a very short intelligence measure that uses only one very specific verbal task. Thus, in the next study, we investigated the influence of a more elaborate measure of cognitive abilities on the detection of collaborative options.

Furthermore, Study 1 dealt with hypothetical behavior only. To extend the scope of the method, we aimed at actual behavior in Study 2 and used an interactive version of the Bamberg Trucking Game. We conducted the study in the laboratory so that participants could actually cooperate instead of merely planning to do so.

## Study 2

In the second study, we tested a computer application of the Bamberg Trucking Game^2^. Dyads of participants interacted in a computer simulation and received monetary reward for delivering goods. The purpose of a behavioral implementation was to study the detection of win–win solutions in actual interactions. It is not to be taken for granted that the same mechanisms are involved in a hypothetical scenario and an interactive setting. We thus aimed to contribute a tool for studying actual behavior in psychological research (cf. Baumeister et al., 2007). Both the behavioral implementation and the monetary reward for delivering goods aimed at increasing the ecological validity of the Bamberg Trucking Game.

As in the first study, our main aim was to estimate the difficulty of finding the win–win solution, this time in actual dyadic interactions. In the dyadic setting, there are factors that may increase or decrease the probability of finding the solution. On the one hand, two parties are involved, and therefore, the probability that at least one of the participants will detect the solution increases. On the other hand, there may be interfering influences that can reduce the probability of finding the solution: Participants may be distracted as they are not only developing a strategy but also in operating the computer game and interacting with the other participant. Thus, we did not have a hypothesis about the difficulty of the game as compared with the scenario-based version.

The second aim of the study was to test whether general mental ability would have an influence on the detection of the win–win solution. On the basis of Study 1, we did not expect that mental ability would predict the detection of the win–win solution. However, to validate the finding, we used a different measure of GMA. We did not see any reason that mental ability would enhance the probability of detecting the solution in a behavioral setting and not in the scenario-based version because the scenario-based version is actually more similar to a task from a cognitive test than the interaction-based implementation.

The dyadic nature of the Bamberg Trucking Game provides the opportunity to distinguish between actor and partner effects (Kenny and Ledermann, 2010). In dyads that succeeded in detecting the win–win solution, one member found the solution, and the other member no longer had the chance to detect the solution. Thus, the partner who did not detect the solution could not be treated like a person in the scenario-based version who did not find the solution or like a member of a dyad that did not find the solution. After all, this partner’s behavior may have helped the other partner to detect the solution. Second, the person might have detected the solution if the other partner had not already detected the solution earlier. Because the distinction between actor and partner exists only in dyads that detected the collaborative solution, the Actor-Partner Interdependence Model (Kenny and Ledermann, 2010) was not applicable. In the following, we will present a strategy that can be applied to analyze the data from the Bamberg Trucking Game to account for this problem.

### Method

#### Sample

Participants were informed that they could earn up to 12 euros and make a minimum of five euros by solving problems. The sample consisted of 112 students (71.4% female) between 18 and 54 years of age (*M* = 23.0, *SD* = 4.4) from the University of Bamberg, Germany. As recordings for one dyad were accidently deleted, one dyad had to be deleted from the final data set, which thus consisted of 110 participants.

#### Procedure

The study consisted of two parts. First, participants completed questionnaires online at home. A few days later, they came to the laboratory to do the interactive task. The online testing included demographic questions, cognitive tasks, and personality questionnaires. Same-sex dyads were randomly assigned and invited to the laboratory.

Upon arriving at the laboratory, participants were greeted and randomly assigned a color (green or yellow). The colors symbolized the colors of the trucks in the game. Participants were given a sticker in their respective color and were asked to stick it to their clothing so their color assignment was visible throughout the study. Participants were seated at one of two identical workplaces with a screen with a 16:9 resolution, a mouse, and a keyboard. Participants faced each other and could not see what was on the other participant’s screen. Participants signed a form in which they agreed not to disclose details about the study and gave permission for their data to be used for scientific purposes.

Participants were told that they would test a logistics simulation. They were given an instruction sheet, explaining the Bamberg Trucking Game (see **Appendix C**). The Bamberg Trucking Game consisted of one practice round and two test rounds. The practice round lasted 2 min and the test rounds 3 min each. The purpose of the practice round was to acquaint participants with the setting and the control panel. In the practice round, participants did not interact. Instead they played a simplified version of the game in which there was only one truck and only one type of loading but two destination fields.

After they completed the practice round, participants had the opportunity to ask the experimenter questions. Then the experimenter placed clip-on microphones onto the participants’ clothing. We used the Apowersoft Free Audio Recorder to record the conversation between the participants during the Trucking Game. Our aim was to obtain data on which of the participants first mentioned the win–win solution (Apowersoft, 2017).

To start the game, both participants had to press the space bar on the keyboards. Before they did so, they had the opportunity to discuss how they would continue. After 3 min, there was a pause, and the game was continued after both participants pressed the space bar. The purpose of the break was for the participants to have the option to discuss changes in strategy. After the second round was over and the Trucking Game was finished, the participants rang a bell, and the experimenter re-entered the room, stopped the audio recordings, and asked the participants to remove the microphones.

Participants then answered a few questions about the study on their computers. First they were asked if they had envisioned strategies that they thought were not possible or not allowed during the transport simulation. They typed their answers into a text box. Afterward, they were asked if they had detected the win–win solution. This question was aimed at identifying participants who had detected the solution but had not shared it with their partner (there was no dyad in which this was the case). Then they were asked if they knew the Trucking Game and what they already knew about it. Finally, participants were asked to provide hypotheses about the purpose of the study.

Finally, participants were paid the money they had earned during the study (3 aaa for participating and 25 cents for each box that had been delivered in the Trucking Game). As we wanted to prevent participants from spreading information about the solution, participants were debriefed via e-mail after the whole study was completed.

#### Measures

##### Intelligence

We used the Short Version of the Hagen Matrices Test (HMT-S; Heydasch et al., 2013) to measure intelligence. The HMT-S consists of two practice matrices and six test matrices. Participants were asked to choose the correct solution from eight possible solutions. They had 2 min to solve each of the six test matrices. The total intelligence score was the number of correctly solved test matrices. The internal consistency was α = 0.58.

##### Detecting the win–win solution

We measured the detection of the win–win solution as a dichotomous variable. The Bamberg Trucking Game software automatically detected instances of players picking up boxes of the opposite color. We obtained the information on success in detecting the win–win solution by first identifying dyads in which there were instances of one player picking up a box of the opposite color. We then analyzed the audio recordings of the conversations before and during the trucking game. The person who first mentioned the possibility of picking up opposite colored boxes was coded as having successfully detected the win–win solution.

Each dyad thus either detected the win–win solution or failed to do so. In the dyads that failed, both members of the dyad were coded as having failed. In successful dyads, we distinguished between the person who detected the solution and the person who did not detect the solution. The person who detected the solution was coded as successful, whereas the other member of the successful dyad was coded as missing. The rationale behind this procedure was that members of successful dyads who did not detect the solution themselves could not be coded as unsuccessful because they could have potentially found the solution later but were deprived of this opportunity after their partner detected the solution. In summary, each participant was coded as unsuccessful, successful, or missing.

### Strategy of Analysis

To test the difficulty of the Bamberg Trucking Game, we calculated the relative frequency of dyads who detected the win–win solution. After one member of a dyad found the solution, the other member no longer had the opportunity to find the solution. Therefore, the unit of analysis was the dyad and not the individual.

A major purpose of the Bamberg Trucking Game was to serve as a method for investigating the influence of personality traits and situational factors on the likelihood of detecting win–win solutions. The dyadic nature allows for the distinction between actor effects (effect of a self-trait on the likelihood of detecting the win–win solution) and partner effects (effect of an other-trait on the likelihood of detecting the win–win solution). The challenge in calculating actor and partner effects is that actors and partners can be distinguished only in successful dyads and not in unsuccessful dyads. In unsuccessful dyads, both members of the dyad are the actor and partner at the same time. This precludes the use of multiple logistic regression analyses in which actor and partner traits are treated as different independent variables. To solve this problem, we used a bootstrapping method. The unit of analysis was the dyad, and the predictors were the actor and partner traits. In cases in which unsuccessful dyads were drawn, one member of the dyad was randomly assigned the role of actor and the other member the role of partner. For every bootstrapped sample, we calculated a multiple logistic regression in which we obtained distributions of estimates for actor and partner effects. On the basis of the distributions, we obtained confidence intervals for the estimates and used these confidence intervals to test the significance of the actor and partner traits.

### Results

A total of 27.3% of all dyads detected the win–win solution in the Bamberg Trucking Game.

To test whether gender predicted the detection of the solution, we calculated a χ^2^ test for 2 × 2 contingency tables. The percentage of the relative frequency of detecting the solution did not differ significantly by gender, χ^2^(1,56) = 0.70, *p* = 0.40. To test whether intelligence (*M* = 4.70, *SD* = 1.30) predicted the detection of the win–win solution, we generated 10,000 bootstrapped samples. We conducted a multiple logistic regression of the detection of the win–win solution on actor intelligence and partner intelligence in each of the samples. Six of the 10,000 analyses failed because they did not converge or reached fitted probabilities of 0 or 1. These cases were excluded from further analyses. The median regression coefficient for the actor intelligence effect was -0.10, whereas the median odds ratio was 0.90. The 95% confidence interval for the regression coefficient of actor intelligence was [-0.69, 0.55], and it was [0.50, 1.73] for the odds ratio. The median regression coefficient for the partner intelligence effect was 0.09, whereas the median odds ratio was 1.09. The confidence interval for the regression coefficient of partner intelligence was [-0.46, 0.86], and it was [0.63, 1.09] for the odds ratio. Both sets of confidence intervals included 0 for the regression coefficients and 1 for the odds ratios, so intelligence did not significantly predict the detection of the win–win solution, nor did it predict enabling the partner to detect the win–win solution.

### Discussion

We successfully implemented the Bamberg Trucking Game as a computer application. Participants were engaged in an actual interaction in which they personally benefited from detecting the win–win solution, that is there was a monetary incentive to deliver as many packages as possible. The actual interaction and the immediate benefit in the form of money arguably increase the ecological validity as compared with the hypothetical scenario.

Detecting the win–win solution was again quite difficult, only 27% of the dyads found the solution. Even though two participants could potentially have an advantage in detecting the solution as compared with the individuals in the scenario-based version, the likelihood that a dyad would detect the solution barely exceeded the likelihood of detecting the solution in the individual task of the scenario-based version. Future research may look for ways to adapt the procedure to make solutions easier and raise detection rates to about 50%.

As expected, we did not find any evidence that general cognitive ability had an impact on either detecting the win–win solution or helping the partner to detect the win–win solution. We replicated the results from Study 1, which showed that general mental ability did not predict the detection of the collaborative solution. These results must be interpreted with caution because we did not control for Type II error probabilities. However, the likelihood that intelligence would have a larger than medium effect size is arguably very low. The result can thus be regarded as further evidence for the divergent validity of the Bamberg Trucking Game. The game does not seem to simply be a cognitive puzzle akin to intelligence test items but might instead specifically focuson mindsets that enable the detection of win–win solutions.

The dyadic nature of the data obtained from the Bamberg Trucking Game allows for the differentiation between actor effects (individual attributes that increase the likelihood of detecting the win–win solution) and partner effects (attributes of the interaction partner that increase a person’s likelihood of detecting the win–win solution). We successfully applied a bootstrap method to calculate the size and significance of actor and partner effects.

## General Discussion

The Bamberg Trucking Game offers two ways of assessing the detection of win–win solutions. The first is a scenario-based version, and the second is an interaction-based version, implemented as a computer application. In contrast to previous scenarios in bargaining or compromise, the Bamberg Trucking game assesses detection of a win–win solution in a situation in which cooperation does not require sacrifice of self-interest and is not related to a risk of the partner defecting. Thus, the paradigm disentangles ability from motivation and aims at assessing whether people are able to detect a win–win solution.

We defined win–win solutions as outcomes involving cooperative behavior in which the outcome is superior to the sum of the possible individual outcomes. We formulated six criteria to describe a paradigm as measuring the detection of an opportunity to collaborate. The Bamberg Trucking Game meets these six criteria: (1) In the Bamberg Trucking Game, the individual gains for both parties applying the win–win solution exceed all possible individual gains from applying any other strategies. (2) The individual gain for each party using the win–win solution exceeds the individual gain derived from a scenario in which the other party was non-existent. Criteria 3, 4, and 5 are theoretically not fully satisfied, but practically they are. The non-compliance of Party A with the win–win strategy is associated with minimal costs to Party B only if Party B goes through with the win–win strategy as compared with another strategy. If one party transports the other party’s goods and this action is not reciprocated, only a little time is lost by picking up the other’s goods and moving the goods from their delivery point to one’s starting point. In any case, there was not a single case of lack of reciprocity in the interaction-based version of the Bamberg Trucking Game. In fact, defection would be immediately obvious to the other party and could be sanctioned instantly, so defection would not be a rational strategy. Criterion 6 is fully satisfied as the win–win solution is not obvious, and only a proportion of participants or dyads detected it in both versions of the Bamberg Trucking Game.

General mental ability was not associated with a higher likelihood of detecting the win–win solution in either the scenario-based version or the interaction-based computer application. This can arguably be regarded as initial evidence for divergent validity because it shows that the Bamberg Trucking Game is not simply a measure of cognitive ability. However, it is important to note that we did not control for Type II errors, and therefore, we cannot conclude that intelligence does not predict the detection of the win–win solution to some degree.

### Limitations

Both versions of the Bamberg Trucking Game are quite difficult. Less than one third of the participants or dyads detected the win–win solution. In the scenario-based version, the frequency was substantially increased when participants were explicitly asked to write down potential solutions that they thought were not allowed in the game.

The interaction-based computer application was complicated to implement. One source of complexity stems from the dyadic nature of the task. Two participants have to be scheduled at the same time to participate in the study. Furthermore, data analysis was also complex. To obtain information on which participant found the win–win solution, we had to analyze audio recordings of the successful dyads. Information on whether the dyads themselves were successful could be obtained from the output files of the Trucking Game application itself. The statistical analysis of the dyadic data was also complex in as far as a non-standardized bootstrapping method was needed to obtain the actor and partner effects.

### Future Research

Because detecting the win–win solution in the Bamberg Tucking Game does not seem to be associated with general mental ability, we suggest that other traits or mindsets may be predictive of finding the solution. The scenario was designed ambiguous so people could interpret it as a zero-sum or a win–win situation. To be able to reframe situations and be flexible in how to interpret a situation may arguably not be the result of a logical thought process but may be more akin to what De Bono (1967) termed *lateral thinking*. The solution is obvious and simple after it is detected and is accompanied by a *eureka effect*. This type of cognitive process has also been termed *insight problem solving* (Weisberg and Alba, 1982; Dow and Mayer, 2004; Ollinger and Knoblich, 2009). Furthermore, emotional intelligence and perspective taking improves relationships (Schröder-Abé and Schütz, 2011). We think it is likely that these traits will foster the finding of win–win solutions and refer to that in the discussion. Thus, we suggest that future research test for whether lateral thinking or insight problem solving predicts the detection of the collaborative solution in the Bamberg Trucking Game.

We introduced the Bamberg Trucking Game as a paradigm that can be applied to assess the detection of win–win solutions. In contrast to public goods games (Hardin, 1968), the prisoner’s dilemma (Rapoport and Chammah, 1965), dictator games (Kahneman et al., 1986), or measures on social-value orientation (Murphy et al., 2011), our approach is not confounded with self-sacrifice, and in contrast to the stag hunt game (Skyrms, 2001) or the prisoner’s dilemma, it is not confounded with the risk of defection by fellow players. Thus, the Bamberg Trucking Game does not measure motivation as the games above do but rather the ability to detect solutions. In contrast to negotiation games (Pruitt and Lewis, 1975; Trötschel et al., 2011), the Bamberg Trucking Game is intuitive and very easy for participants to understand, and the solution is not merely pareto-optimal but also individually optimal.

Future research should investigate the relationship between the detection of the win–win solution in the Bamberg Trucking Game and these methods. We hypothesize that detecting the win–win solution in the Bamberg Trucking Game is related to cooperating in the stag hunt game and the negotiation game but not related to prosocial behavior in public goods games, the prisoner’s dilemma, or the dictator game or to social-value orientation. In the stag hunt game, the scenario is a potential win–win situation, but non-compliance with the win–win strategy by the partner leads to a zero payoff. In the Bamberg Trucking Game, non-compliance by one party barely decreases the payoff for the other party compared with the compromising strategy. The negotiation games are designed in such a way that valuable items for one person are of negligible value to the other and vice versa, thus making the optimal solution very similar to a win–win solution. Neither the public goods game, the prisoner’s dilemma, nor the dictator game assess win–win solutions as defined in the present approach because, in these games, higher individual outcomes are associated with fewer payoffs for others—they represent zero-sum scenarios. Thus, we argue that only the Bamberg Trucking Game assesses the actual detection of win–win solutions.

The Bamberg Trucking Game scenario could become the first item in a psychometric test, which assesses the ability to detect win–win solutions as an individual difference variable. Thus future research could develop more ambiguous scenarios in which win–win solutions can be. The ability to detect win–win solutions then could be modeled as a latent variable.

### Conclusion

The Bamberg Trucking Game is a valuable paradigm for research on cooperation, win–win solutions, and mutually beneficial behavior. It is a paradigm that can be applied to assess whether people are able to find a win–win solution in a seemingly conflict-prone situation or a potentially conflict-evoking dilemma. Its added value lies in its characteristics of being unconfounded with self-sacrifice, risks, or the opportunity to free-ride. Rather than assessing prosocial attitudes, it assesses the ability to actually detect a win–win solution in an ambiguous situation.

Win–win solutions are ways of increasing the benefit of everyone involved as compared with the sum of the outcomes of all individual endeavors. The tool can be used for research on situational and individual factors that predict the finding of win–win solutions, that is, the paradigm can be used to compare actors who differ in various traits (e.g., agreeableness, creativity, or empathy) and test whether there is an effect on whether a mutually beneficial solution is identified. Similarly, the paradigm can be used to test which situational factors (e.g., supervisor encouragement or group building measures) are conducive to the finding of win–win solutions.

After such predictors have been found, they can be useful for training and selecting people for positions in organizations in which it is crucial to have a keen eye for cooperative options. In fact, many real-life situations can at first be understood as zero-sum games but with the appropriate mindset can be recognized as situations where mutually beneficial action is possible. The Bamberg Trucking Game can not only be used for assessment but could also be used as a training tool in showing people how a situation that may not suggest collaboration at first sight can be reframed to be a win–win situation. Because of the importance of collaboration in human interactions we think the game may be helpful in research, assessment, and training.

## Ethics Statement

All subjects gave written informed consent in accordance with the Declaration of Helsinki. The protocol was approved by the Ethics Committee of the University of Bamberg.

## Author Contributions

DN: conception and design, analysis and interpretation of data, and drafting of the paper. AS: conception and design, interpretation of data, and revision of the paper. AP: design and programming and acquisition of data.

## Conflict of Interest Statement

The authors declare that the research was conducted in the absence of any commercial or financial relationships that could be construed as a potential conflict of interest.

## Footnotes

1 An example of such an instruction for the optimal strategy would be: “Call driver B at the very beginning and agree with her/him on the following strategy: Both drivers transport both their own and each other’s goods. Driver A starts by taking SR and only uses SR from then on, while driver B takes LR1 or LR2 the first two times and will then also start taking only SR, as driver A will be able to drive right behind driver B after that. Thus, as soon as both drivers are on the side of SB/DA, both continue to deliver both kinds of goods with driver A following driver B on SR.”

2 The source code for the game is available upon request.

## Appendix A

### Instructions for the scenario-based version of the Bamberg Trucking Game

Imagine that you own a logistics firm. Your task is to deliver as many goods from your starting point (SA) to your delivery point (DA) as quickly as possible. You have at your disposal a truck along with a driver (A). Another logistics firm has the goal to transfer as many goods as possible from their starting point (SB) to their delivery point (DB). That firm also has a truck (B) at their disposal. It does **not matter how the goods are taken to the delivery points (DA and DB);** it matters only that **as many goods as possible are delivered as quickly as possible.**

#### Important

• You can carry only one package of goods at a time.
• In the middle of the area, you can see a short road (SR). This is a single-lane road, which means it is not wide enough for two trucks to pass each other.
• Besides the short road, there are two longer roads (LR1 and LR2). They are **50%** longer than the short road (SR), which means it takes longer to use them than the short one.
• At any point in time, your driver is allowed to **call the driver of Truck B and communicate with him or her**.
• Except for the limitations described in the rules, you are **free to do whatever you want**.

Before your driver sets off to deliver the goods, you can give him or her very specific instructions on how to act. Please explain in the text box below the instructions that you would give your driver. It is not important that you write down word for word what you would say but that you express the main message. It is important that your driver knows exactly what he or she should do on the basis of your instructions. Once again: Your goal is to deliver as many goods as possible in as little time as possible to your delivery point.

## Appendix B

### Instructions for the second page of the scenario-based version of the Bamberg Trucking Game

Some people told us that they had thoughts about how to solve the problem but did not mention them because they thought they were not compatible with the rules of the game. If you envisioned solutions that you abandoned because you thought they are not allowed, please write them in the text box below.

## Appendix C

### Instructions for the computer simulation of the Bamberg Trucking Game^3^

#### Instruction trucking simulation

The goal of the trucking simulation is to transport and deliver as many boxes as possible within 3 min. You are steering the yellow ball  (i.e., your truck), and the other participant is steering the green ball . For every yellow box  that is deposited in the yellow destination field , you will receive 25 cents. The other participant will receive 25 cents for every green box  that is deposited in the green destination field . You can **pick up only one box at a time**.

To steer the truck, please use the arrow keys on your keyboard .

The boxes can be picked up and put down by using the **space bar**.

In the lower right corner of your screen, you can see how many yellow boxes have been delivered so far. In the upper left corner, you can see how many green boxes have been delivered so far.

You can move around the entire grey field. Please consider: the **road** in the middle is narrow, which means that two trucks cannot pass each other.

You have 3 min to complete the first round. At the top of your screen, you can see the time left until the end of the round. During the simulation, you can communicate with the other participant. The conversation will be recorded.

After the first round, there will be a break. During the break, you can also communicate. Please press the **spacebar** when you wish to continue the simulation. As soon as both participants have pressed the spacebar, the second round will begin. The second round will also last 3 min.

Before starting, you will have the opportunity to acquaint yourself with the control panel in a practice round. The practice round will last 2 min. In that round, you will act alone so that you can move around freely and try everything out.

If you have further questions, please address them to the experimenter. If you understand everything, please tell the experimenter that you wish to start the practice round.
